# Local anaesthetics or their combination with morphine and/or magnesium sulphate are toxic for equine chondrocytes and synoviocytes in vitro

**DOI:** 10.1186/s12917-017-1244-8

**Published:** 2017-11-07

**Authors:** L. M. Rubio-Martínez, E. Rioja, M. Castro Martins, S. Wipawee, P. Clegg, M. J. Peffers

**Affiliations:** 1 Institute of Veterinary Science, University of Liverpool, Leahurst Campus, Chester High Road, CH647TE Neston, UK; 2 Faculty of Veterinary Science, Rajamangala University of Technology Srivijaya (Thailand) and Institute of Aging and Chronic Disease, University of Liverpool, Liverpool, UK; 3 Institute of Aging and Chronic Disease, University of Liverpool, Liverpool, UK

**Keywords:** Local anaesthetic, Morphine, Magnesium sulphate, Chondrocyte, Synoviocyte, Equine

## Abstract

**Background:**

Chondrotoxic effects of local anaesthetics are well reported in humans and some animal species but knowledge on their toxic effects on synoviocytes or equine chondrocytes or the effects on cellular production of inflammatory cytokines is limited. The purpose of this study was to evaluate the in vitro effects of local anaesthetics, morphine, magnesium sulphate (MgSO_4_) or their combinations on cell viability and pro-inflammatory cytokine gene expression of equine synoviocytes and chondrocytes.

Equine synoviocytes and cartilage explants harvested from normal joints in a co-culture system were exposed to mepivacaine (4.4 mg/ml), bupivacaine (2.2 mg/ml), morphine (2.85 mg/ml) and MgSO_4_ (37 mg/ml) alone or each local anaesthetic plus morphine or MgSO_4_ or both together. Chondrocyte and synoviocyte cell viability was assessed by CellTiter-Glo Luminescent Cell Viability Assay. Synoviocyte gene expression of IL-1β, IL-6 or TNF-α was measured and compared using the ∆∆CT method.

**Results:**

Morphine alone, MgSO_4_ alone or their combination did not alter cell viability or the expression of IL-1β, IL-6 or TNF-α. However, local anaesthetics alone or in combination with morphine and/or MgSO_4_ reduced cell viability and increased the gene expression of IL-1β, IL-6 or TNF-α. Single short exposure to local anaesthetics is toxic to both chondrocytes and synoviocytes and their combination with morphine and/or MgSO_4_ enhanced the cytotoxic effects.

**Conclusions:**

This in vitro study gives further evidence of the absence of cytotoxic effects of morphine alone, MgSO_4_ alone or their combination on normal articular tissues. However, local anaesthetics alone or in combination with morphine and/or MgSO_4_ have cytotoxic effects on equine articular tissues.

**Electronic supplementary material:**

The online version of this article (10.1186/s12917-017-1244-8) contains supplementary material, which is available to authorized users.

## Background

Intra-articular injections of local anaesthetics are commonly performed in humans and horses to determine sources of pain and as perioperative pain control [1]. Despite their widespread use, there is growing concern over the potential toxicity of these substances and their long-term effects on articular tissue [2, 3]. Chondrotoxic properties of local anaesthetic agents have been reported in humans and animals [2, 4–6], but knowledge of their effect on equine chondrocytes is limited [7, 8]. The majority of these studies have investigated their effects on chondrocyte viability, but the effects of local anaesthetics on synoviocytes are still largely unknown. The synovium contributes to nociceptive, inflammatory and degradative responses and therefore it is vital that the effects of intra-articular injections are also studied on the synovium. Recent studies on rabbits and dogs suggest that the toxic effects of local anaesthetic on synoviocytes may affect the onset of chondrolysis associated with intra-articular use of local anaesthetics [9–11].

Because of the local anaesthetic related chondrotoxic effects, alternatives for articular analgesia are being sought in humans [3]. Morphine is an opioid that provides excellent articular analgesic and anti-inflammatory effects when administered intra-articularly in humans [12, 13] with apparently minimal toxic effects on human and canine chondrocytes [2, 14]. Intra-articular administration of morphine causes analgesia, and reduces swelling and synovial inflammatory markers in horses [15–18], although it was associated with release of large molecular weight proteoglycans into the synovial fluid [19]. Magnesium sulphate (MgSO_4_) is routinely administered intra-articularly to human patients for peri-operative analgesia [20] and does not cause a significant reduction in human chondrocyte viability [21]. Moreover, addition of MgSO_4_ to local anaesthetics reduced the toxic effects of the latter on human chondrocytes in vitro [22]; and intra-articular administration of MgSO_4_ attenuated the development of osteoarthritis (OA) in a rat model [23].

We hypothesised that local anaesthetics but not morphine or MgSO_4_, would produce deleterious effects on chondrocyte and synoviocyte viability and increase the expression of pro-inflammatory cytokines. We further hypothesised that morphine or MgSO_4_ in combination with a local anaesthetic would prevent the negative effects exerted by local anaesthetics alone.

## Methods

The aim of this study was to evaluate the in vitro effects of clinically-relevant doses of local anaesthetics, morphine, MgSO_4_ or their combinations on equine chondrocyte and synoviocyte viability and gene expression of pro-inflammatory cytokines in a co-culture in vitro model. We hypothesised that local anaesthetics would produce deleterious effects on chondrocyte and synoviocyte viability and increase the expression of pro-inflammatory cytokines. We further hypothesised that morphine and/or MgSO_4_ in combination with a local anaesthetic would reduce the impact of the negative effects exerted by local anaesthetics alone on cell viability and gene expression of pro-inflammatory cytokines.

### Study design

An experimental in vitro study was performed on equine synoviocytes and cartilage explants harvested from normal joints and using a co-culture system.

### Tissue sample collection

Tissue samples were obtained from metacarpophalangeal joints of 10 skeletally mature horses (6–10 years old) from an abattoir within 5 h of slaughter. Synoviocytes were obtained from three horses and articular cartilage samples obtained from seven different horses. Only horses with grossly normal joints were included in the study.

Under sterile conditions, synovial membrane was harvested from the entire synovial surface of the joint and synoviocytes isolated as previously described [24]. Synoviocytes were cultured in Dulbecco’s Modified Eagle Medium (DMEM) (Sigma-Aldrich, Darmstadt, Germany) supplemented with 10% foetal calf serum (FCS), 100 units/ml penicillin, 100 μg/ml streptomycin, L-glutamine 4 mM (all from Invitrogen, Paisley, UK) and 500 ng/ml amphotericin B (Bio Whittaker, Lonza, San Diego, California, USA), in routine laboratory conditions (37 °C, 5% O_2_) until 90% confluent. After treatment with 0.05% w/v trypsin, synoviocytes were passaged and cultured until 90% confluent. Synoviocytes (passage 2) were then frozen in 10% DMSO in DMEM complete (DMEM supplemented as above) and stored in liquid nitrogen. Full thickness cartilage was collected from the entire surface of the distal condylar area of the third metacarpus. Cartilage was diced into explants of approximately 2 mm x 2 mm, mixed and placed in complete DMEM complete at 37 °C, 5% O_2_ for 18 h to equilibrate.

Synoviocytes from three horses were thawed in a water bath at 37 °C for 1 min, mixed and suspended in DMEM complete to a final concentration of 50,000 live cells per ml. Synoviocytes were plated in 24-well plates at a concentration of 50,000 cells per well (2 wells per group). After equilibration at 37 °C, 5% O_2_ for 24 h, trans-well inserts (0.45 μm pore, 12 mm; Millicell Cell Culture Inserts PIHA01250; Sigma-Aldrich, Darmstadt, Germany) were located into the wells and cartilage explants from 7 horses were placed separately into the inserts (5 explants per insert) in duplicate. The co-culture system was maintained in an incubator at 37 °C, 5% O_2_ for 24 h at which time co-cultures were exposed to different treatments for 2 h (Table 1). Drugs were diluted in DMEM complete to a total volume of 2 ml per well to achieve the following final concentrations: mepivacaine 4.4 mg/ml, bupivacaine 2.2 mg/ml, morphine 2.85 mg/ml, magnesium sulphate 37 mg/ml. Concentrations were based on the average volume of synovial fluid in the equine metacarpophalangeal joint (12.5 mL) [25] and the doses commonly used in clinical practice [16, 17, 26–29]. Exposure time (2 h) was based on the reported half-life for the local anaesthetics in horses [26, 30]. After exposure, the medium containing the different treatments was removed and replaced by DMEM complete and cultured for additional 48 h. At this time synoviocytes and explants were harvested. Cartilage explants were snap-frozen in liquid nitrogen and stored at -80 °C. Synoviocytes from the same group were released from the wells using 250 μL Tri-reagent (Sigma-Aldrich, Darmstadt, Germany) per well for 30 min and immediately stored at -80 °C

**Table 1:** Treatment groups

Group	Treatment
1	Control
2	Mepivacaine only
3	Mepivacaine + morphine
4	Mepivacaine + MgSO4
5	Mepivacaine + morphine + MgSO4
6	Morphine only
7	MgSO4 only
8	Morphine + MgSO4
9	Bupivacaine only
10	Bupivacaine + morphine
11	Bupivacaine + MgSO4
12	Bupivacaine + morphine + MgSO4

Treatment groups to which synoviocyte-articular cartilage explant co-cultures were exposed. Concentrations used were mepivacaine 4.4 mg/ml, bupivacaine 2.2 mg/ml, morphine 2.85 mg/ml and magnesium sulphate 37 mg/ml diluted in DMEM complete to a total volume of 2 ml per well

### Cell viability

Cell viability was performed on tissues from one well per group. Chondrocytes were recovered after digesting cartilage explants with 1 mg/ml collagenase II (Worthington Biochemical Corporation, Lakewood, US). Cell viability analysis was performed on synoviocytes and chondrocytes with CellTiter-Glo Luminescent Cell Viability Assay using a Glomax Detection System as per manufacturer instructions (Promega, Wisconsin, US). This assay determines the number of viable cells in culture based on quantification of adenosine triphosphate by luminescence, which signals the presence of metabolically active cells [31, 32].

### RNA extraction and Real-time Quantitative Polymerase Chain Reaction (qPCR) analysis

RNA was extracted from cartilage explants and synoviocytes as previously described [33]. Total RNA concentration was determined using a Nanodrop-2000c (Thermo Fisher Scientific). Complimentary DNA (cDNA) was prepared [33]. Real-time qPCR was used to measure relative gene expression for markers of three pro-inflammatory cytokines: Interleukin 1 beta (IL1β), Interleukin 6 (IL6) and Tumour Necrosis Factor alpha (TNFα) compared to the reference gene glyceraldehydes-3-phosphatedhyrogenase (GAPDH) as described previously using SYBR Green technology [33]. Primer sequences are listed in Table 2.

**Table 2:** Primer sequences of the reference gene (GAPDH) and pro-inflammatory cytokines IL1β, IL6 and TNFα used in the study

Gene	GeneBank Accession	Direction	Primer Sequence (5′-3′)
GAPDH	NM_001163856	Forward	GCATCGTGGAGGGACTCA
		Reverse	GCCACATCTTCCCAGAGG
IL-1β	NM_001317261	Forward	GCCTAAGAATACTACATCCAGAGA
		Reverse	GGCATTGATTAGACAACAGTGAA
IL-6	NM_001082496	Forward	CTGCTCCTCGTGATGGCTAC
		Reverse	CCGAGGATGTACTTAATGTGCTG
TNF-α	NM_001081819	Forward	CCTTCCACTCAATCAACCCTCT
		Reverse	CACGCCCACTCAGCCACT

Primer sequences of the reference gene (GAPDH) and proinflammatory cytokines IL1β, IL6 and TNFα used in the study

### Statistical analysis

Data were analysed using commercially available statistical software (SPSS, version 22.0, 2013, Chicago, USA). Graphical displays and Anderson-Darling test were used to check for departures from assumptions of normality. Log transformations were applied when data were non-normally distributed. Gene expression data were normalised to the reference gene using the ∆∆CT method [34] and statistical analysis applied to the 2^-∆CT values. General linear models with Dunnet’s comparisons with control group were performed. Significance was considered when *p* < 0.05. Gene expression results were normalised to the control group.

## Results

### Cell viability

**Fig. 1:**
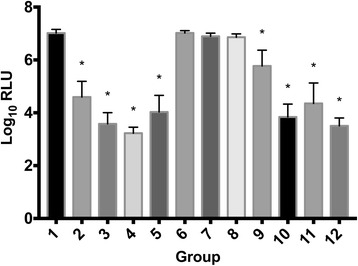
Synoviocyte viability Cell viability measured as luminescence signal (shown as log10 transformed Relative Light Units [RLU]) for synoviocytes exposed to different treatments (for further information on treatment groups refer to Table 1). Error bars indicate 95% confidence interval. Asterisk (*) indicates significant difference (*p* < 0.05) with group 1 (control). Groups included are: control (group 1); mepivacaine (group 2); mepivacaine + morphine (group 3); mepivacaine + MgSO_4_ (group 4); mepivacaine + morphine + MgSO_4_ (group 5); morphine (group 6); MgSO_4_ (group 7); morphine + MgSO_4_ (group 8); bupivacaine (group 9); bupivacaine + morphine (group 10); bupivacaine + MgSO_4_ (group 11); and bupivacaine + morphine + MgSO_4_ (group 12)

**Fig. 2:**
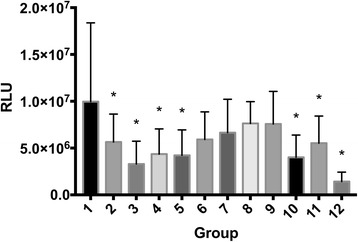
Chondrocyte viability Cell viability measured as luminescence signal (mean values of Relative Light Units [RLU]) for chondrocytes exposed to different treatment groups (for further information on treatment groups refer to Table 1). Error bars indicate 95% confidence interval. Asterisk (*) indicates significant difference (*p* < 0.05) with group 1 (control). Groups included are: control (group 1); mepivacaine (group 2); mepivacaine + morphine (group 3); mepivacaine + MgSO_4_ (group 4); mepivacaine + morphine + MgSO_4_ (group 5); morphine (group 6); MgSO_4_ (group 7); morphine + MgSO_4_ (group 8); bupivacaine (group 9); bupivacaine + morphine (group 10); bupivacaine + MgSO_4_ (group 11); and bupivacaine + morphine + MgSO_4_ (group 12)

Raw data of cell vaiability are provided in Additional file 1. The effects of different treatments on synoviocyte and chondrocyte viability are presented in Figs. 1 and 2 respectively. Compared with control group, chondrocyte or synoviocyte viability did not change in groups exposed to morphine alone, MgSO_4_ alone or morphine and MgSO_4_ in combination. Chondrocyte viability was significantly reduced in chondrocytes exposed mepivacaine alone or in combination with morphine and/or MgSO_4_ (groups 2, 3, 4 and 5). Exposure to bupivacaine alone did not reduce chondrocyte viability in comparison with control group; however, chondrocyte viability was significantly reduced in groups exposed to combinations of bupivacaine with either morphine or MgSO_4_ or both combines (groups 10, 11 and 12). Synoviocyte viability was reduced in all groups exposed to either local anaesthetic (mepivacaine or bupivacaine), and any of their combinations with morphine and/or MgSO_4_. (groups 2, 3, 4, 5, 9, 10, 11 and 12).

### Real-time qPCR

**Fig. 3:**
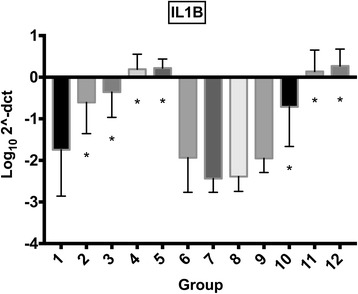
Synoviocyte IL1β gene expression IL1β gene expression (mean log_10_2^-ΔCT) for synoviocytes exposed to different treatment groups (for further information on treatment groups refer to Table 1). Error bars indicate 95% confidence interval. Asterisk (*) indicates significant difference (*p* < 0.05) with group 1 (control). Groups included are: control (group 1); mepivacaine (group 2); mepivacaine + morphine (group 3); mepivacaine + MgSO_4_ (group 4); mepivacaine + morphine + MgSO_4_ (group 5); morphine (group 6); MgSO_4_ (group 7); morphine + MgSO_4_ (group 8); bupivacaine (group 9); bupivacaine + morphine (group 10); bupivacaine + MgSO_4_ (group 11); and bupivacaine + morphine + MgSO_4_ (group 12).

**Fig. 4:**
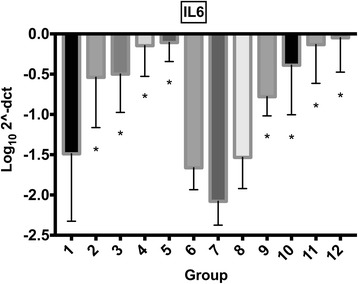
Synoviocyte IL6 gene expression IL6 gene expression (mean log_10_2^-ΔCT) for synoviocytes exposed to different treatment groups (for further information on treatment groups refer to Table 1). Error bars indicate 95% confidence interval. Asterisk (*) indicates significant difference (*p* < 0.05) with group 1 (control). Groups included are: control (group 1); mepivacaine (group 2); mepivacaine + morphine (group 3); mepivacaine + MgSO_4_ (group 4); mepivacaine + morphine + MgSO_4_ (group 5); morphine (group 6); MgSO_4_ (group 7); morphine + MgSO_4_ (group 8); bupivacaine (group 9); bupivacaine + morphine (group 10); bupivacaine + MgSO_4_ (group 11); and bupivacaine + morphine + MgSO_4_ (group 12).

**Fig. 5:**
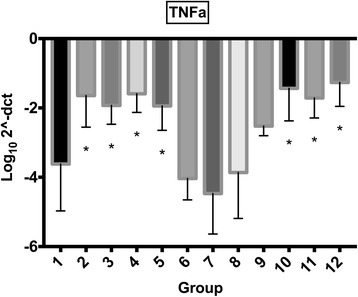
Synoviocyte TNFα gene expression (mean log_10_2^-ΔCT) for synoviocytes exposed to different treatment groups (for further information on treatment groups refer to Table 1). Error bars indicate 95% confidence interval. Asterisk (*) indicates significant difference (*p* < 0.05) with group 1 (control). Groups included are: control (group 1); mepivacaine (group 2); mepivacaine + morphine (group 3); mepivacaine + MgSO_4_ (group 4); mepivacaine + morphine + MgSO_4_ (group 5); morphine (group 6); MgSO_4_ (group 7); morphine + MgSO_4_ (group 8); bupivacaine (group 9); bupivacaine + morphine (group 10); bupivacaine + MgSO_4_ (group 11); and bupivacaine + morphine + MgSO_4_ (group 12)

Raw data on gene expression are provided in Additional file 1. Treatment with morphine, MgSO_4_ or their combination did not have an effect on synoviocyte gene expression of IL-1β, IL-6 or TNF-α. Exposure to mepivacaine alone (group 2) increased expression of IL-1 β, IL6 and TNF-α, while exposure to bupivacaine alone (group 9) only significantly increased expression of IL6. Combination of either mepivacaine or bupivacaine with morphine, MgSO_4_, or both (groups 3, 4, 5, 10, 11 and 12) increased expression of IL-1β, IL-6 and TNF-α (Figs. 3, 4, 5). Gene expression from cartilage explants did not yield consistent amount of RNA for downstream qRT-PCR.

## Discussion

Chondrotoxic effects of local anaesthetics are reported in the literature and results of this study confirm that a single short exposure to local anaesthetics causes significant articular cytotoxic effects characterised by decreased viability of both equine synoviocytes and chondrocytes. These effects were not counteracted by the addition of morphine and/or MgSO_4_. Except for the mepivacaine effect on TNF-α expression, local anaesthetics alone, as well as morphine alone, MgSO_4_ alone, or morphine and MgSO_4_ combined, did not increase the expression of pro-inflammatory cytokines by synoviocytes; however, the pro-inflammatory cytokines expression were increased when local anaesthetics were combined with morphine and/or MgSO_4_.

The present study demonstrates that bupivacaine at a concentration of 0.22% is harmful for articular equine synoviocytes but its negative effects are less pronounced on chondrocytes. A protective effect from the existence of intact extracellular matrix on chondrocytes from the cartilage explant has been previously suggested [6, 14], although this was recently questioned on an experimental canine model [10]. Local anaesthetics have drug-, dose- and time-dependent cytotoxic effects [5, 10, 35–37]. Bupivacaine 0.0625% did not cause cell death in canine cartilage and synovium explants [10] and varying toxicity has been observed for bupivacaine concentrations in the range of 0.125–0.25% [2, 5, 10, 35]. Bupivacaine concentrations ≥0.5% have consistently been reported to exert toxic effects on human and animal chondrocytes [5, 35], including horses [7] but interestingly did not affect the viability of rabbit type B synoviocytes in monolayer [11]. Different species sensitivities, methodologies or cell lines may explain different results between studies. Mepivacaine is less frequently administered as an intra-articular analgesic in people and there are fewer studies investigating its effects on articular cells. In contrast to our study, mepivacaine was less toxic than bupivacaine on human [35] and equine [7] chondrocytes on monolayer cultures. However, the bupivacaine concentration used in the present study was lower than in previous studies and has been previously associated with chondrotoxic effects on human cartilage [35] and equine chondrocytes [38]. Mepivacaine is commonly administered intra-articularly in horses at doses similar to that in the present study and temporary synovitis has occasionally been reported [27].

Few studies have investigated the effects of local anaesthetics on the production of inflammatory molecules by articular tissues. Following exposure to bupivacaine a decrease in nitric oxide and PGE_2_ concentrations was observed in an IL-1-treated co-culture explant model; and that was suggested to be the result of the lack of viable cells remaining after the exposure [2]. However, in our study increased expression of IL1-β, IL6 or TNF-α was observed despite decreased viability

Intra-articular administration of morphine or MgSO_4_ produces analgesia without clinical evidence of adverse effects in people [12, 13, 20, 39–41]. The present study corroborates the absence of deleterious effects of morphine or MgSO_4_ on canine human and canine chondrocyte viability in vitro [2, 14, 21, 22], and extends this absence of toxicity to synoviocytes. Furthermore, the combination of morphine and MgSO_4_ did not have negative effects on cell viability, which warrants investigation of the potential analgesic effect of the combination *in vivo*. In addition, no effects on expression of pro-inflammatory cytokines were observed when tissues were exposed to morphine and/or MgSO_4_. Anti-inflammatory effects of opioids have been reported after systemic or local administration. Morphine exposure of IL-1-treated cartilage explants decreased nitric oxide and PGE_2_ production [2]. Intra-articular administration of morphine reduced nucleated cell count in synovial fluid of chronic arthritis patients [42] and serum amyloid A and total protein levels in horses with experimentally-induced synovitis [15]. Anti-inflammatory effects of magnesium have also been recognised. Low magnesium promotes inflammation and up-regulates IL1α and IL6 production in endothelial cells [43] while magnesium supplementation attenuated the development of OA [23] and rheumatoid arthritis (RA) [44]. MgSO_4_ acts on *N*-methyl-D-aspartate receptors and although the present study did not include an OA or RA model, *N*-methyl-D-aspartate receptors involved in the development of OA and RA are also present in normal human and mouse articular chondrocytes [45]. Results of this study support the clinical use of morphine and warrant further investigation on MgSO_4_ as intra-articular analgesic drugs in horses and other species.

Addition of MgSO_4_ to local anaesthetics counteracted harmful effects on human chondrocyte viability in vitro [22] and we aimed to determine if the same was true in a synoviocyte and cartilage explant co-culture model. However, the addition of morphine, MgSO_4_ or both to mepivacaine or bupivacaine did not counteract cell death caused by mepivacaine and furthermore, decreased chondrocyte viability in comparison with the control group when combined with bupivacaine. Combination of either local anaesthetic with morphine and/or MgSO_4_ also enhanced the synoviocyte expression of IL1β, IL6 and TNFα. The reasons for the enhanced toxicity of the drug combinations are unclear. The mechanisms of action of local anaesthetics, morphine and MgSO_4_ differ. The cytotoxic effects of local anaesthetics are related to a concentration-dependent mitochondrial depolarization with subsequent alteration in transmembrane potential and cell death [46]. Osmolality-related cytotoxicity has been observed with high concentrations of magnesium (500 mg/ml) [22] but the concentration used in the present study was 37 mg/ml. Interaction between molecules or their combination with the culture medium or changes in the pH of the environment may be involved [47]. A time-dependent increased chondrotoxicity has been observed for the combination of corticosteroid and local anaesthetics [48]. The pH of the treatment solutions in the present study were nor determined and buffer solutions were not used; however, low pH was not the cause for decreased viability of bovine chondrocytes [1, 49] and addition of buffering solution can increase chondrotoxicity [47]. Chemical incompatibility between the anaesthetic solution and culture medium leading to crystal formation and high cell death rates has been suggested by some authors [1, 7]; however, precipitation was not observed in the present or other studies [2, 10, 38]. Although combination of local anaesthetics and MgSO_4_ are being administered clinically into joints without reported clinical side effects [20, 50] further research into the chemical compatibility or interactions between drugs is warranted.

The present study has a number of limitations. A co-culture equine model was used to allow evaluations of both cartilage and synovium components as many inflammatory mediators and analgesic receptors are found in the synovium, and synoviocytes have important roles in maintaining articular health and participating in pathophysiological processes [51, 52]. However, results from this in vitro study may not be the same *in vivo*. Use of synovial explants [2, 10] could have enhanced the system although recent studies support the comparability of explants and monolayer cultures to assess cytotoxicity [2, 10]. The preferential type of synoviocyte present in the culture is uncertain and a mixture of both macrophage-like and fibroblast-like synoviocytes is suspected in this study as they were frozen at passage 2 [52]. Drug concentrations and duration of exposure were based on clinically used doses, joint volume and pharmacokinetics in horses. However, the drug concentrations remained constant during the 2 h exposure, which could be interpreted as a relative overdose as clearance of the drug is expected to occur already within this time in the live animal. The horse is an accepted translational models of naturally-occurring osteoarthritis [53] but drug effects can differ between species. Normal articular tissues were used and more severe chondrotoxic effects are expected on degenerative versus normal cartilage [35]. The reasons for the poor RNA extraction from cartilage explants are uncertain. This methodology has been used successfully in other studies but equine mature articular cartilage is relatively acellular and we used small size explants. Previous studies in our lab have yielded 1 μg of RNA from the cartilage harvested from both third metacarpal condyles in adult horses (Peffers M.J. Unpublished data). The use of isolated chondrocytes, larger size or higher number of cartilage explants, or use of a different methodology may have yielded gene expression data from the cartilage explants. Quantification of adenosine triphosphate by luminescence has been shown reliable method to assess cell viability [14, 31].

## Conclusions

The present study corroborate the toxic effects of single short exposure to local anaesthetics on both equine chondrocytes and synoviocytes using an in vitro co-culture model, with more severe effects observed for mepivacaine than bupivacaine. It also provides evidence that synoviocytes respond to drugs commonly administered intra-articularly, which warrants further investigation. The present study indicates that morphine and MgSO_4_ are not toxic to synoviocytes when exposed either alone or in combination, which supports the clinical use of morphine and warrants further investigation of MgSO_4_ as intra-articular analgesic drugs in horses and other species. However, further investigation into drug incompatibilities is required as combinations of either mepivacaine or bupivacaine with morphine, MgSO_4_ or both did not alleviate any detrimental effects from the local anaesthetics, but actually caused cell death and increased expression of pro-inflammatory enzymes.

## Additional file

Additional file 1: Raw data on chondrocyte and synoviocyte viability and synoviocyte gene expression. (XLSX 18 kb)

## Abbreviations

cDNA: Complimentary deoxyribonucleic acid
DMSO: Dimethyl sulfoxide
FCS: Foetal calf Serum
GAPDH: Glyceraldehyde-3-phosphatedhyrogenase
IL-1β: Interleukin 1β
IL-6: Interleukin 6
MgSO_4_: Magnesium sulphate
OA: Osteoarthritis
qRT-PCR: Real-time quantitative polymerase chain reaction
TNF-α: Tumour necrosis factor α
